# Diabetes care provided by national standards can improve patients' self‐management skills: A qualitative study of how people with type 2 diabetes perceive primary diabetes care

**DOI:** 10.1111/hex.13247

**Published:** 2021-03-28

**Authors:** Rebecka Husdal, Eva Thors Adolfsson, Janeth Leksell, Lena Nordgren

**Affiliations:** ^1^ Department of Public Health and Caring Sciences Family Medicine and Preventive Medicine Uppsala University Uppsala Sweden; ^2^ Centre for Clinical Research Västmanland Uppsala University Västerås Sweden; ^3^ Department of Medical Sciences Clinical Diabetology and Metabolism Uppsala University Uppsala Sweden; ^4^ School of Education, Health and Social Studies Dalarna University Falun Sweden; ^5^ Centre for Clinical Research Sörmland Uppsala University Eskilstuna Sweden

**Keywords:** continuity, primary diabetes care, qualitative study, self‐management, type 2 diabetes mellitus

## Abstract

**Background:**

The increasing incidence of type 2 diabetes mellitus [T2DM] has resulted in extensive research into the characteristics of successful primary diabetes care. Even if self‐management support and continuity are increasingly recognized as important, there is still a need for deeper understanding of how patients' experiences of continuity of care coincide with their needs for self‐management and/or self‐management support.

**Objective:**

To gain a deeper understanding of how people with T2DM perceive Swedish primary diabetes care and self‐management support.

**Methods:**

This qualitative study used focus groups as the means for data collection. Participants were identified through a purposive sampling method differing in age, sex, diabetes duration and latest registered glycated haemoglobin level. Twenty‐eight participants formed five focus groups. Qualitative content analysis was applied to interview transcripts.

**Results:**

The main theme emerging from the focus group data was that diabetes care provided by national standards improved self‐management skills. Two themes that emerged from the analysis were (a) the importance of a clarification of structures and procedures in primary diabetes care and (b) health‐care staff ‘being there’ and providing support enables trust and co‐operation to enhance self‐management.

**Conclusions:**

Individual patients' self‐management resources are strengthened if the importance of providing relational continuity, management continuity and informational continuity is considered. Patients also need assistance on ‘how’ self‐management activities should be performed.

**Patient contribution:**

Prior to the study, one pilot focus group was conducted with patients to obtain their perspectives on the content of the planned focus groups; thus, patients were involved in both planning and conduct of the study.

## 1 INTRODUCTION

Worldwide, the number of people with type 2 diabetes mellitus (T2DM) is increasing. About 5% of the total Swedish population has diabetes, which is comparable to the figures in, for example, Australia^1^ and the UK.^2^ In these countries, and many others, T2DM represents about 90% of all diabetes cases.^1^, ^2^, ^3^ Though it is well known that adequate glycaemic control can reduce the risks for complications and premature death in people with diabetes, many patients struggle to follow recommendations for treatment.^4^ Consequently, they remain at risk for diabetic complications.

In order to achieve glycaemic goals while maintaining good health, people with T2DM need daily self‐management.^5^ Self‐management can be defined as *‘the individual's ability to manage the symptoms, treatment, physical and psychosocial consequences and life style changes inherent in living with a chronic condition’* ^6^ (p. 178). However, self‐management can be complicated and requires confidence on the part of the patient as well as support from health‐care professionals.^7^ In Sweden, the National Board of Health and Welfare is responsible for national guidelines for diabetes care. The aim of the guidelines is to offer good and equal diabetes care for the entire population. National guidelines for diabetes care provide recommendations, 140 recommendations in adults with diabetes. The guidelines cover the following areas: prevention and lifestyle, glucose control, cardiovascular disease, nursing, diabetes complications and pregnancy and diabetes.^8^ Most people with T2DM in Sweden will be treated in primary care by general practitioners (GP) and registered nurses (RN) educated in diabetes care.^8^, ^9^

The increasing incidence of T2DM has resulted in extensive research into the characteristics of successful primary diabetes care.^10^, ^11^, ^12^ However, identifying these characteristics has proven to be a daunting task due to the complexity of the T2DM disease.^13^, ^14^ Furthermore, the development and organization of primary diabetes care have been found to be guided by the perspectives of health‐care professionals rather than those of the patients.^15^ In a meta‐synthesis, Brundisini et al^16^ found that even though health‐care professionals and patients had a common understanding of barriers to and facilitators of medication adherence, many sources of misunderstanding—such as lacking communication and/or overlooked occasions for intervention—remained. In addition, recent qualitative studies have repeatedly reported that patients with T2DM have unmet needs regarding self‐management skills and support and that more patient‐centred and individualized support is needed.^17^, ^18^, ^19^, ^20^, ^21^, ^22^, ^23^, ^24^, ^25^, ^26^, ^27^, ^28^, ^29^, ^30^, ^31^ This tends to result in a gap between the expectations of people with T2DM and the care they receive.^30^, ^32^

One important aspect of care quality is continuity of care, which in turn has been shown to be associated with better glycaemic control.^33^ Haggerty et al^34^ defined continuity as: *‘the degree to which a series of discrete healthcare events is experienced as coherent and connected and consistent with the patient's medical needs and personal context’* (p. 1221). The same authors identified three types of continuity: relational continuity, management continuity and informational continuity.^34^

A recent Swedish report about the general public's view of continuity within primary care^35^ stated that health‐care administrations, organizations and processes need to take peoples' differing needs and preferences into account, to a higher degree than they currently do. In the report, it was suggested that a range of different solutions would be needed if the health‐care organization was to meet the general public's differing needs and preferences. It was also considered important to develop knowledge about how peoples' needs and preferences can be met at both local and individual levels. Such knowledge can lead to clarified goals and development of strategies for meeting the general public's many needs. The report also mentioned that members of the general public value different aspects of care differently and that preferences can be related to specific situations that will change over time.^35^

There is also another important difference that should not be ignored—that between continuity in the delivery of care, that is the care providers' perspective, and continuity in the experience of care, that is the patients' perspective.^36^ These days, patients' views and experiences are highly valued when quality of care is measured or evaluated. Even if self‐management support is increasingly recognized as important, there is still a need for deeper understanding of how patients and health‐care providers can be more deeply engaged in productive interactions with the mutual goal of improving patients' health.^15^ However, it is not clear how patients' experiences of continuity of care coincide with their needs for self‐management and/or self‐management support. Thus, the aim of this study was to gain a deeper understanding of how people with T2DM perceive Swedish primary diabetes care and self‐management support.

## 2 METHODS

### 2.1 Study design and setting

In this study, a qualitative research design was used. Data were collected in 2019. The report has been made with a basis in the consolidated criteria for reporting qualitative research checklist.^37^ The setting was five primary care centres in a region of mid‐Sweden. The inclusion of primary care centres was based on their different list sizes and geographical areas (ie urban and rural areas). The primary care centres were required to have a manager and staff among whom at least half of the GPs were specialized in general medicine and half of the RNs were a specialist RN in primary health care.^38^ All five primary care centres had at least one diabetes nurse employed who were responsible for patients with T2DM. In Sweden, there are two levels of diabetes nurses. A diabetes nurse means an RN with at least 15 ECTS (European Credits Transfer Accumulation System), advanced level, within diabetes care. A specialist RN in diabetes care takes an examination within advanced level degree of 60 ECTS, ‘Diabetes Care ‐ Specialist Nursing Programme’.

### 2.2 Study participants

A purposive sampling method was used to identify participants who differed in age, sex, duration of diabetes and latest registered glycated haemoglobin level (ie HbA1c, the average blood glucose level during the preceding 6‐8 weeks), in order to obtain a sample resembling a typical patient group at a primary care centre. Information used for inclusion and exclusion was retrieved from medical records. People with T2DM were eligible if they were at least 18 years of age and had been diagnosed with T2DM for a minimum of 1 year. People with T2DM were ineligible if they did not speak or understand Swedish, received residential care in a nursing home or were diagnosed with any kind of dementia.

To achieve maximum variation, a total of 150 potential participants were identified, using the criteria above, from among the listed patients at the five primary care centres (30 people with T2DM from each centre). The medical records provided information based upon which 16 individuals were excluded (ie no contact information [n = 5], changed primary care centre [n = 1], diagnosed with type 1 diabetes [n = 2], in residential care [n = 5], dementia [n = 3]). Invitation letters and information about the study's aim were sent by post to the people with T2DM confirmed as eligible (n = 134).

The first (RH) and the second author (ETA) contacted potential participants over a 2‐week recruitment period to ask about participation and to agree upon a time and place for the focus group. Thirty‐two people with T2DM agreed to participate. However, two participants withdrew due to illness or private reasons and two missed the focus group. The final sample consisted of 28 people with T2DM who each took part in one of the five focus groups, with 5‐6 people in each group.

### 2.3 Data collection

Face‐to‐face focus groups were used as the means for data collection. The choice of using focus groups rather than individual interviews was made to shed light on the research question from different perspectives through in‐depth discussions between people with T2DM. In addition, this would provide an opportunity for patients to discuss and share their individual experiences with each other.

Prior to the study, one pilot focus group was conducted with patients in order to obtain their perspectives on the content of the planned focus groups; thus, patients were involved in both planning and conduct of the study. The pilot group patients did not participate in the five planned focus groups. The pilot focus group view led to minor changes in how the questions were worded, to better suit the participants; for example, words like ‘self‐management’ were replaced.

The focus groups were conducted at the respective primary care centres where the people with T2DM were listed. The focus groups varied in length, between 60 and 90 minutes, and were conducted by a moderator (ETA) and an assistant moderator (RH). None of the authors had a prior relationship to any of the participants.

Before the focus groups started, the participants were given information on the aim of the study, the reason for conducting it and their ethical rights. The interview guide was based on previous studies.^9^, ^14^, ^39^ The participants were asked to describe their experiences of contacts with primary care, resources and organizational features, as well as their experiences of support for self‐management. Situation‐based probes such as ‘what do you mean (…)’ and ‘tell me more about (…)’ were used to gain deeper understanding of the participants' descriptions. At the end of each focus group, the moderator summarized the discussions and the participants had the opportunity to comment on the summary and give clarifications if something had been misunderstood or was unclear.

### 2.4 Data analysis

All focus groups were audio‐recorded and transcribed verbatim. Transcripts were verified against the audio recordings. The data were analysed using qualitative content analysis.^40^ The analysis started with the second (ETA) and third author (JL) reading through the transcribed focus groups line by line. All transcripts were read several times, and relevant parts were extracted in order to gain an overall picture. The first part of the analysis process was to extract meaning units from the transcripts. Then, the meaning units were condensed to a description close to the text. Interpretation of the condensed meaning units led to identification of underlying meanings, which were each labelled with a code. The identified codes were grouped together based on similarities and differences and sorted into themes and subthemes that reflected the latent contents of the focus groups. After a process that included reflection and discussion of themes and subthemes, an overall theme was formulated. To strengthen the credibility of the analysis, the entire research group analysed the codes and proposals for themes and subthemes. Next, all authors jointly reflected on the evolving results to capture the entirety of the data material. The research group reflected and discussed until consensus was reached. To promote credibility, quotes have been used below in the presentation of the results.

### 2.5 Ethics

The regional ethics committee in Uppsala approved the study (dnr: 2019‐02033). The results are presented in a way that ensures none of the participants can be identified. All participants were informed, in writing and verbally, that participation was voluntary. Informed consent was obtained from all participants before participation. In addition, participants were informed that they could end their participation at any time.

## 3 RESULTS

### 3.1 Participants characteristics

A total of 28 people diagnosed with T2DM for at least 1 year were interviewed. Table 1 presents demographic information for the sample. There was an even sex distribution among the participants and they had been diagnosed with diabetes for a mean of 9 years. Most participants were married. Twenty participants were retired.

**TABLE 1:** Characteristics of study participants

Characteristics	Number (n = 28)
Age (years), mean	67
Sex	
Male	14
Female	14
Duration of diabetes (years), mean	9
HbA1c (mmol/mol), mean	58
Marital status	
Single	8
Married/registered partner	17
Living apart	3
Educational level	
≤9 y	12
10‐12 y	9
College/university	6
Occupational status	
Working	6
Long‐term sick leave (>3 mo)	1
Retired	20
Taking care of the household	1
Number of visits at primary care centre in the preceding year, mean	2

### 3.2 Themes

**FIGURE 1:**
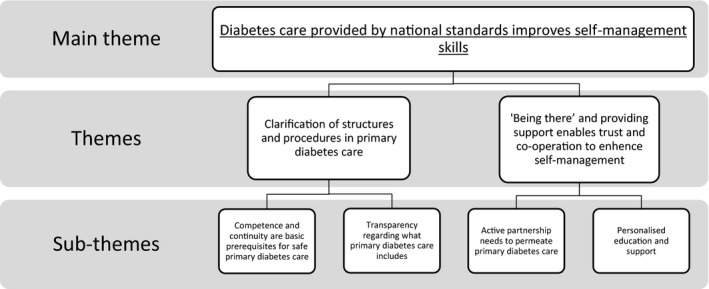
Main theme, themes and subthemes based upon data from five focus groups

From the analysis of the latent content in the five focus groups, a main theme was gleaned: ‘*Diabetes care provided by national standards improves self‐management skills*’. In other words, diabetes care based on national guidelines improves self‐management skills. The main theme was based on two themes and four subthemes (see Figure 1).

#### 3.2.1 Clarification of structures and procedures in primary diabetes care

This theme was based upon two subthemes: ‘*Competence and continuity are basic prerequisites for safe primary diabetes care*’ and ‘*Transparency regarding what primary diabetes care includes*’.

##### Competence and continuity are basic prerequisites for safe primary diabetes care

The contents of the first subtheme indicated that the participants perceived basic prerequisites for safe diabetes care to be that staff had adequate competence in diabetes care, that there was a continuity in their visits and that they were able to feel trust for staff. Some participants said that competence was more important than continuity.

For me, it doesn't really matter who I see, as long as they have the knowledge … So I won't get someone who doesn't say anything and next time I'll get someone who is very well‐read … then the difference becomes huge. Who the person in question is, it doesn't bother me who it is. (FG4; A)

On the other hand, the participants perceived that lack of competence or continuity could create a sense of uncertainty. The uncertainty contributed to lower reliance on one's own ability, as well as on the staff's competence. In addition, lack of continuity contributed to staff giving different messages, which further reinforced the sense of uncertainty.

There's a mix of highly competent people and absolute greenhorns and so … I really don't know who's who … and that gives a sense of insecurity … Should I trust myself or should I trust someone else or … (FG4; EL)

Other participants stated that the meaning of continuity was having a single professional contact, which was seen as a prerequisite for adherence to the guidelines. A suggestion that was given during the focus groups was that patients should regularly receive a questionnaire with questions about their health. They argued that it could serve as a way to be continuously followed up.

Yeah, but I think it would create more confidence too, if it was just like having a programme in one's care plan, that you send out this survey, like stuff that you want to know from us, every four months, or how they can keep in touch … that's continuous … so that you feel the contact isn't lost. (FG4; A)

##### Transparency regarding what primary diabetes care includes

The second subtheme was based upon the participants' views regarding getting a clear description of the contents of health‐care visits, who had responsibility for coordination with other health‐care providers and individual‐based digitization. The participants said that with clarity regarding the content in care meetings, they would gain a better picture of what was expected of them. The participants suggested that a checklist should be used, to help them know for example which diabetes‐related controls would be included in a visit.

I haven't seen anyone by the book, sort of. It's different each time. It's not like one of those checklists … (FG4; O)

You should have like a note about what's included so you could tick it off yourself. (FG1; S)

The responsibility for coordination was especially important if patients had several problems that required contact with multiple health‐care professionals.

Yeah, there's no one else who takes care of you, so you have to take care of it yourself. There's no coordination with other medical units, you have to take care of that yourself. (FG4; J)

As regards digitization, it was said that individual circumstances and prerequisites such as knowledge and experiences should be considered.

You could have, like, a page for conversation. You log on to my page, and my information and medications are there, and the dose I have, and then doctors can … if I want a new prescription I can go there and the doctor can change my dose and send a message like ‘I want you to take 10 units instead of …’, like that. (FG3; M)

Some participants had a routine of reading their medical records online, while others had no experience and knowledge of how they logged in to their electronic medical records.

#### 3.2.2 ‘Being there’ and providing support enable trust and co‐operation to enhance self‐management

This theme was based on following subthemes: ‘*Active partnership needs to permeate primary diabetes care*’ and ‘*Personalized education and support*’.

##### Active partnership needs to permeate primary diabetes care

The participants described a need for active partnership in diabetes care and that it was a shared task to support the individual patient in diabetes self‐management.

So, the view, if it was shared, that it is a shared task to fix me, or the way that care is conducted, then of course that could matter. But that probably requires that one works somewhat differently with the care organisation at a regional level, I think. (FG4; EL)

The participants described that they wanted to be seen and acknowledged as human beings. It was something they expressed as being the heart of adherence to how care should be conducted.

The doctor behaved ‘by the book’, and one thing he did … he looked at me when he spoke and talked to me, some doctors just sit there and take notes in the medical record at the same time and ‘yes, hmm yes’ and ‘then I'll write this to you’, yeah … like, ‘do you understand that’ … He looked at me and he talked to me. (FG3; M)

There were also participants who mentioned that life changes continuously and that it is important that the medical staff see this and are attentive on one's changing needs.

… this … life … a lot of stuff happens … If you becomes mentally stressed, the values go up … maybe you would need a counsellor or a psychologist or something like that, that could somehow get you to think differently. It's hard when you end up in this darkness to sort of find your way out, and you still have to function. (FG2; N)

There were participants who said that they were in control of their self‐management, while others felt that they lacked tools or needed support to achieve the conditions for independent self‐management.

For me, I try to take control and if I see that it's going wrong then you have to act, maybe first with medications and checking the values, and then to call and get an appointment in order to discuss this. (FG2; G)

One participant reflected on motivating leadership—becoming a leader who leads him‐ or herself.

Something a doctor maybe should think about, motivating leadership … that I can become a good leader if I am able to lead myself. But something must motivate me, you'll find reasons to motivate yourself, to take this into account and check your values and so on. But not everyone does, and then maybe another motivating leader has to step in, perhaps the doctor who says ‘see how great it feels when you have lowered it by 10%’ or whatever you've done. (FG4; EL)

##### Personalized education and support

Participants pointed out that caregivers needed to identify a patient's level of knowledge regarding diabetes care and that it should not be taken for granted that a patient had knowledge. This was seen as crucial. Participants argued that health‐care providers should provide them with evidence‐based information and not just make things easy for themselves and refer to other sources.

The diabetes nurse said that ‘you can google a bit and read a bit about the medications from home’. Then I felt that I would have liked to have gotten a brochure or something. It doesn't feel good to google it … It feels like a lot of responsibility is placed on me. I want this information to be classified, I don't want to sit there and google it. Now I usually go to 1177 [note: a Swedish online healthcare guide] and read up, but I don't really feel that it's acceptable. (FG3; M)

The participants also highlighted a need for alternative ways of obtaining information and education, as each patient is unique, takes in information and learns in their own way. Information and learning need to be provided on an on‐going basis, as there are always new research results and technologies.

I can envision that you could get information letters by e‐mail or something like that when there are new findings. (FG5; L)

I think you could have information meetings when something new has happened. If it's the case that it's once a year or every five years or … to have one when something has changed that is of general interest … (FG4; J)

It was clear that participants needed support in the form of concrete advice on how knowledge could be applied in different situations, so‐called ‘how to’. The participants spoke of ‘groping in the dark’ and not knowing how to move forward.

And how do you do that? They could put a bit more energy into talking about that. (FG4; M)

I go for a walk twice a day and I try to keep my weight down and not gain weight, but still I don't seem to lower my long‐term sugar and that can make me desperate, because eventually I don't know what to do other than just take more pills. (FG2; GB)

The participants believed that they needed support to be motivated to make changes in order to achieve treatment goals.

These are the benefits … that they provide motivation. Not the other way around: now you have stuck holes in your fingers and the results didn't improve. It's the wrong way around and there's no motivation in that. (FG4; EL)

Furthermore, they said they were not helped by being accused of doing things wrong. They felt that together with the caregiver they could find solutions that would motivate them.

It has been, like … try to achieve these goals … not that I have to … more like an appeal: ‘try to reach these because then you'll feel better’ … they ask, ‘do you think you can drop this much?’ … There isn't, like, any more pressure to do it. (FG3;O)

## 4 DISCUSSION

The analysis revealed an overall theme that described how people with T2DM perceived Swedish primary diabetes care and self‐management support. The main theme was that diabetes care provided by national standards improved self‐management skills. Further, the analysis revealed two themes: (a) the importance of a clarification of structures and procedures in primary diabetes care and (b) that health‐care staff ‘being there’ and providing support enables trust and co‐operation to enhance self‐management.

The findings add to previous knowledge that both patients and health‐care professionals need to be encouraged to engage in productive interactions in daily diabetes primary care, in order to strengthen and improve patients' self‐management skills.^15^ The participants in this study appeared to be rather content with the general structure and procedures in the primary diabetes care received. However, there were some unmet needs that needed to be addressed. These unmet needs might indicate that interactions with health‐care providers were insufficient concerning management continuity, relational continuity and informational continuity. This finding is consistent with previous research that describes a gap between the expectations of people with T2DM and the care they receive.^30^, ^32^

The results also showed that the participants' needs for continuity of care involved different aspects of continuity, such as continuously meeting with the same health‐care professional and continuously learning about their condition. The participants also perceived that health‐care professionals could support their confidence in self‐management by ‘being there’. This result can be interpreted in relation to the definition of continuity of care given by Haggerty et al.^34^ In relation to management continuity, unmet needs were described regarding more individualized support for independent self‐management. The experience of being provided with information and advice concerning the condition, but not being told how activities should be performed, is consistent with a previous study by Aweko et al.^17^ Experiences like this can lead to struggling and confusion.^17^ It is important that health‐care professionals incorporate practical recommendations when they act to support patients' self‐management skills. Even though studies on self‐care and self‐management have been published since the 1990s^6^ and self‐management is a critical cornerstone in the treatment of diabetes, people with T2DM still experience an unmet need of receiving practical recommendations from health‐care professionals. Commonly, health‐care professionals provide advice about self‐management. However, as suggested by Barlow et al,^6^ greater use of peer education might be more helpful for patients' self‐management abilities—and more cost‐effective. Thus, health education for peers should be accommodated to a higher degree than today in the self‐management programmes within primary diabetes care.

The participants described that in order to improve self‐management activities, there was a need for a ‘collective investment’ in the patient‐professional relationship, encouraging a patient to become ‘a leader who leads him‐/herself’. In line with the findings of Kristensen et al,^26^ relational continuity was highly valued by the participants in this study. Relational continuity, then, should be a top priority as regards patients with complex chronic conditions and impaired self‐management ability. Already in 2002, Norris et al^5^ pointed out that contact time is the only significant predictor of improved glycaemic control. Thus, to maintain improved glycaemic control, long‐term interventions are required and health‐care professionals need to spend sufficient time with their patients.

In relation to informational continuity, the participants expressed a permeating sense that primary diabetes care required more personalized support. At the same time, however, the participants expressed a need for a checklist, to fully understand both the contents and the purpose of their appointments. This creates a daunting task: providing individualized care, while structuring it in accordance with a checklist would require a high level of pedagogical ability and competence among health‐care providers. Burridge et al^23^ found that patient change is a difficult process, and even when patients know how to perform self‐management activities, they do not automatically act in line with their knowledge. The authors^23^ thought the cause for this could be that some health‐care professionals still used older models for health education that meant they provided information and expected patients to change as a result thereof. However, Burridge et al^23^ also acknowledged existing gaps between knowing and changing and between changing and sustaining change, which was also emphasized by the participants in this study. Thus, patients and professionals need to engage in an active partnership that means they work together. Moreover, the partnership needs to accommodate both the health‐care professionals' expert biomedical knowledge and the patients' expert contextual knowledge of living with diabetes.^23^ This is in line with what was expressed by the participants in this study.

There are some limitations to this study that need to be addressed. It is possible that people with a strong desire to report barriers were more willing to participate in the study. Thus, the findings cannot be generalized to all people with T2DM living in Sweden. A risk of using focus groups instead of individual interviews is that the participants might not have experienced a permissive environment characterized by openness, allowing them to speak freely, and might have had a sense of being judged by others. However, these limitations were balanced by the study's strengths. At the beginning of the focus group, all participants were informed that the discussion was confidential and would not affect their future care or treatment. Another strength was that the study included participants from five different primary care centres, resulting in a variation of experiences from primary diabetes care. To gain satisfactory breadth and depth, we also included individuals with different characteristics. In addition, all four authors participated in the final analysis, to reduce subjective interpretation of the focus group—and the use of quotes enables assessment of the credibility of this qualitative study. Also, a benefit of conducting the qualitative content analysis as described by Graneheim and Lundman^40^ is that it provides a structure to relate to the manifest texts and to promote identification of latent themes. Another strength of the study is that the moderator (ETA) is a registered dietician, with training in diabetes care and experience of conducting focus groups with people with T2DM. Thus, the potential risk of missing certain aspects was minimized. Neither the moderator (ETA) nor the assistant moderator (RH) had any care or personal relationships with the participants, which may have meant that the participants spoke more freely about their experiences.

## 5 CONCLUSIONS

This study has identified that in order for people with T2DM to improve their self‐management skills, they would need health‐care professionals to provide diabetes care by national standards. On the basis of these findings, some practical steps for future work in primary diabetes care can be suggested. First, in order to better understand and strengthen the individual patient's self‐management resources, health‐care professionals need to provide relational continuity, management continuity and informational continuity. Second, even when health‐care professionals adequately inform patients about self‐management activities, the patients still need assistance on ‘how’ these activities should be performed, preferably based on national guidelines for diabetes care. Furthermore, future research should focus on how to bring about change at system level, so that GPs and RNs in primary diabetes care are adequately resourced to improve the support to their T2DM patients.

## CONFLICT OF INTEREST

The authors have no conflicts of interest to declare.

## Data Availability

The data that support the findings of this study are available from the corresponding author upon reasonable request due to privacy/ethical restrictions.
